# Total thyroidectomy as primary surgical management for thyroid disease: surgical therapy experience from 5559 thyroidectomies in a less-developed region

**DOI:** 10.1186/s12957-016-0772-1

**Published:** 2016-01-22

**Authors:** Jisheng Hu, Nan Zhao, Rui Kong, Dawei Wang, Bei Sun, Lifeng Wu

**Affiliations:** 1 Department of Pancreatic and Biliary Surgery, First Affiliated Hospital of Harbin Medical University, 23 Youzheng Street, Nangang District, Harbin, 150001 Heilongjiang Province China; 2 Department of Respiratory Medicine, First Affiliated Hospital of Harbin Medical University, 23 Youzheng Street, Nangang District, Harbin, 150001 Heilongjiang Province China; 3 Department of Critical Care Medicine, Harbin Medical University Cancer Hospital, 150 Haping Street, Nangang District, Harbin, 150081 Heilongjiang Province China

**Keywords:** Total thyroidectomy, Subtotal thyroidectomy, Papillary thyroid carcinoma, Thyroid surgery, Hypoparathyroidism, Recurrent laryngeal nerve injury

## Abstract

**Background:**

The objective of this study was to evaluate the safety of total thyroidectomy for thyroid disorders and summarise the treatment experience in a less-developed region.

**Methods:**

This was a retrospective observational cohort study using the computerised database of the First Affiliated Hospital of Harbin Medical University. All consecutive thyroidectomy patients from 2003 to 2014 were included in this study. Demographics, surgical procedure, diagnoses, morbidity and mortality were retrospectively reviewed.

**Results:**

There were a total of 714 men and 4845 women in this study, with a mean age of 55 (range 9–87) years. A total of 4632 patients underwent total thyroidectomy for primary surgical treatment, and 189 patients previously underwent partial thyroidectomy. A total of 56.2 % of the patients had multinodular goitre, including 12.23 % who were thyrotoxic. Graves’ disease and Hashimoto’s disease were diagnosed in 2.82 and 7.23 % of the patients, respectively. Papillary thyroid cancer was identified in 1336 patients, 44.99 % of whom had papillary microcarcinoma. The total prevalence of permanent complications of first-time and secondary surgeries was 0.35 and 7.41 %, respectively. During thyroid surgery, 945 patients underwent parathyroid autotransplantation.

**Conclusions:**

Initial total thyroidectomy can be safely performed for both benign and malignant thyroid diseases in a less-developed region. The morbidity of a secondary surgical procedure after subtotal thyroidectomy is significantly high compared to first-time surgery.

## Background

In recent years, the incidence of thyroid disorders, including thyroid malignancy, has increased rapidly; however, the reasons why the incidence of thyroid diseases is increasing substantially are not yet completely understood. Routine physical check-ups, particularly the widespread application of high-resolution ultrasonography, may explain this trend [1]. Moreover, iodine deficiency diseases (IDDs) are widespread in China, and Heilongjiang province is one of the IDD-endemic areas. After the nationwide introduction of a mandatory universal salt iodisation (USI) programme in 1996, IDDs may have been effectively controlled [2]. Because of the higher iodine concentration in edible salt in our country than the WHO-recommended standard and because of the local eating habits, a high-salt diet may lead to excessive iodine intake. However, the excessive iodine intake problems caused by the Chinese USI programme have not yet been taken seriously [3] and may account for the increasing incidence of thyroid diseases [3, 4]. Total thyroidectomy (TT) is undoubtedly the optimal surgical treatment for high-risk thyroid cancer; however, the rationality of this surgical approach for the treatment of benign multinodular goitre, toxic multinodular goitre and Graves’ disease and even for the treatment of low-risk well-differentiated thyroid carcinomas remains controversial [5–9]. The disequilibrium of medical resources makes increasing numbers of patients from less-developed areas become concentrated in a large hospital such as ours. For patients here, the cost to cure their disorders ranges from a month or even a year’s income, and many patients do not return for regular re-examination because of economic factors. The objective of this study was to determine whether TT is an effective, safe and appropriate procedure to manage thyroid diseases and provide insight into the management of thyroid diseases in economically less-developed areas.

## Methods

A retrospective review was conducted to all patients undergoing thyroid surgery between 2003 and 2014 at the First Affiliated Hospital of Harbin Medical University. All clinical and pathological data were entered prospectively into a computerised database. We extracted data on the sex, age, nature of thyroid disease, incidence of postoperative complications and final pathology from the departmental medical records. The Medical Ethics Committee at First Affiliated Hospital of Harbin Medical University (Harbin, Heilongjiang, China) approved this study, and all patients signed informed consents.

### Preoperative evaluation

Preoperative evaluation in all patients included serum thyroid hormones, thyroid antibodies, parathyroid hormone (PTH), calcium, indirect laryngoscopy and neck ultrasonography. For patients with thyroid nodules, fine needle aspiration is the most appropriate examination to provide a rapid and accurate assessment. Very aggressive cancers, such as poorly differentiated and anaplastic thyroid cancers, which may invade local structures, require deliberate planning. Computed tomography (CT) is also performed in all patients to provide important adjunct anatomical information about the thyroid as well as related structures in the neck, and it is a necessary preoperative imaging study that affects both the extent of surgery and method of anaesthesia induction [10–12]. All thyrotoxic patients received pharmacologic therapy preoperatively, such as beta-blockers, which were continued until 10–14 days after the surgery to prevent perioperative thyroid crisis. TT was performed for patients with thyroid nodules involving both lobes, Graves’ disease and toxic goitre in our institution.

### Surgical procedure

After the induction of anaesthesia, the neck is gently extended with a cushion under the shoulders. Surgery begins with the design of incisions, which should balance incision aesthetics and adequate exposure for safe surgery. A collar incision two fingerbreadths above the clavicle within or parallel to the skin line will not only assure a minimal incision scar but also provide sufficient exposure. Routine local anaesthesia was used to facilitate a free skin flap and alleviate pain postoperatively. The anterior cervical muscle group was routinely divided lengthways, and the thyroid gland was dissected on the plane of the capsule, with careful ligation of all vessels. Both recurrent laryngeal nerves (RLNs) were exposed routinely during the course of the dissection, and intraoperative electrophysiological nerve integrity monitoring was not used in our procedures. Every attempt was made to identify and preserve the parathyroid glands in situ. Parathyroid glands that could not be preserved with an adequate blood supply were meticulously dissected from the thyroid gland, diced and autotransplanted in the sternocleidomastoid muscle at the end of the operation. This technique is widely practiced and is an efficient method of avoiding long-term hypoparathyroidism. No drainage tube or suction drainage with negative pressure was needed when only hemithyroidectomy (HT) or TT with/without central neck dissection was completed. When lateral neck dissection was performed, a drainage tube was required in case of lymphatic or chylous leak.

### Postoperative management and postoperative follow-up

Lifelong treatment of levothyroxine replacement was required at a dose of 100–200 μg per day, depending on weight and sex, and serum TSH was followed up to adjust the dose of levothyroxine. Routine 2-week oral calcium and vitamin D supplementation (Caltrate D, Wyeth, USA, containing 1.5 g calcium carbonate and vitamin D_3_ 125 IU in each tablet) without laboratory assessment was administered to all patients who had total thyroidectomy, and this method is a safe and cost-effective for preventing symptomatic hypocalcaemia [13, 14]. Patients take 3 g of calcium three times daily for the first week and 1.5 g three times daily for the second week. If symptoms of hypocalcaemia remain, additional doses of 2 g of calcium gluconate are intravenously injected. Hypoparathyroidism was considered permanent if a calcium supplement was still required after 6 months. In patients with hoarseness, an additional indirect laryngoscopy was scheduled at 1, 3 and 6 months after surgery or until the vocal cord function recovered. After 6 months, we considered RLN palsy to be permanent. Based on the patient’s physical condition and will, patients of TT, HT and TT + CND were usually discharged within 2–3 days after surgery. Patients who underwent modified neck section were not discharged until the drainage tube was removed, which occurred when the drainage fluid was less than 25 ml for three consecutive days. Patients with Horner’s syndrome, chylous leakage, severe dyspnoea causing tracheomalacia or RLN palsy and haemorrhage had delayed discharge.

## Results

A total of 5559 thyroid operations were performed between 2003 and 2014. There were 714 men and 4845 women with a mean age of 52 (range 9–87) years. The basic characteristics of the patients and the stage of patients with malignant diseases are presented in Table 1. All patients underwent thyroidectomy, as a first-time surgery in 5370 patients and as a secondary thyroid surgery in 189 patients. Of the reoperations, 138 previously underwent subtotal thyroidectomy (ST) for multinodular goitre and required a complete thyroidectomy for recurrent diseases. In addition to the thyroid surgery, 217 patients had central neck dissection and 108 had modified neck dissection. HT was performed initially for 413 patients with a solitary nodule or multiple lesions within one lobe, and all removed tissue was revealed as benign by intraoperative frozen pathological section. A total of 51 patients required a HT for recurrent disease (Table 2). A total of 945 patients underwent parathyroid autotransplantation, and none suffered from permanent hypoparathyroidism. Of the 945 patients, 1302 parathyroid glands were autotransplanted and 102 were intrathyroidal parathyroid.

**Table 1:** Characteristics of 5559 patients and stages of malignant thyroid cancer

Sex ratio (M/F)	714:4845
Mean age (years)	55 (9–87)
Preoperative diagnosis	
Euthyroid	4324
Bilateral	3540
Unilateral	685
Isthmus	99
Hyperthyroid	674
Diffuse goitre	177
With thyroid nodule(s)	497
Hypothyroid with thyroid nodules	601
Stage of malignant thyroid cancer^a^	
Total malignant thyroid disease	1640
Patient older than 45 years	1295 (78.96 %)
I	844 (51.46 %)
II	411 (25.06 %)
III	264 (16.10 %)
IV	121 (7.38 %)

^a^Staging according to the seventh edition of AJCC Cancer Staging Manual 2010

**Table 2:** Complications of the various thyroid operations

	First-time surgery (*n* = 5370)					Revision surgery (*n* = 189)		
Complication	TT (4632)	HT (413)	TT + CND (217)	TT + MND (108)	Total	CT after ST (138)	LT after HT (51)	Total
RLN injury no. (%)								
Temporary	29 (0.63)	4 (0.10)	8 (3.69)	4 (3.70)	45 (0.84)	19 (13.77)	2 (3.92)	21 (11.11)
Permanent	15 (0.32)	0 (0)	2 (0.92)	1 (0.93)	18 (0.34)	11 (7.97)	0 (0)	11 (5.82)
Total	44	4	10	5	63	30	2	32
Hypoparathyroidism no. (%)								
Temporary	152 (3.28)	9 (2.18)	19 (8.76)	10 (9.26)	190 (3.53)	25 (18.12)	1 (1.96)	26 (13.76)
Permanent	1 (0.02)	0 (0)	0	1 (0.02)	1 (0.02)	3 (2.17)	0 (0)	3 (1.59)
Total	153	9	19	191	191	28	1	29

*TT* total thyroidectomy, *HT* hemithyroidectomy, *CND* central neck dissection, *MND* modified neck dissection, *CT* completion thyroidectomy, *LT* lobectomy, *ST* subtotal thyroidectomy, *RLN* recurrent laryngeal nerve

Table 3 summarises the postoperative histopathological diagnosis based on paraffin section examination of the resected thyroid glands. Of these, 56.2 % of the patients had multinodular goitre, including 12.23 % who were thyrotoxic. Graves’ disease and Hashimoto’s disease were diagnosed in 2.82 and 7.23 % of the patients, respectively. Papillary thyroid cancer was identified in 1336 patients, 44.99 % of whom had papillary microcarcinoma.

**Table 3:** Postoperative histopathological diagnosis and associated complications

Diagnosis	No.	Percent
Benign	3919	70.50
Multinodular goitre	3124	56.20
Toxic	382	6.87
Non-toxic	2766	49.76
Graves’ disease	157	2.82
Hashimoto’s disease	402	7.23
Subacute thyroiditis	85	1.53
Follicular adenoma	107	1.92
Malignant	1640	29.50
Papillary thyroid carcinoma	1336	24.03
Microcarcinoma	601	10.81
With Hashimoto’s disease	297	5.34
With Graves’ disease	43	0.77
Follicular carcinoma	130	2.34
Medullary carcinoma	65	1.17
Anaplastic carcinoma	4	0.07
Other	104	1.87
Total	5559	100

Table 2 also shows the complications of the various thyroid operations, and the incidence of postoperative complications was compared between patients with different approaches. There was no mortality in the postoperative period. Haemorrhage, a serious complication requiring reoperation, only occurred in a 38-year-old female patient who underwent TT with modified neck dissection. For the first-time surgery, permanent RLN injury occurred in 18 patients, including three cases of nonrecurrent laryngeal nerve and 9 cases of tumour invasion. Furthermore, during secondary completion thyroidectomy after ST, we could not successfully expose the RLN in 34 patients as a consequence of fibroblasts and scar tissue. Of these, 10 had unilateral permanent RLN palsy and 1 patient had bilateral permanent RLN palsy. The incidence of permanent RLN injury combined with permanent hypoparathyroidism for the first-time surgery was 0.35 %, and the permanent complication rate of the secondary surgery was 7.41 %, including both completion thyroidectomy after ST and LT (lobectomy) after HT. Moreover, the relationship between the grade of goitre and the complication rates of initial TT is shown in Table 4. The grading system is as follows: a goitre that can be touched but cannot be seen is grade I; a goitre that can be both touched and seen but is not beyond the exterior margin of the sternocleidomastoid muscle is grade II; and a goitre that can be both touched and seen and is beyond the exterior margin of sternocleidomastoid muscle is grade III.

**Table 4:** Relationship between grade of goitre and complication rates of initial TT

	Grade of goitre (*n* = 4632)			
Complication	0 (*n* = 1302)	I (*n* = 2063)	II (*n* = 828)	III (*n* = 439)
RLN injury				
Temporary	2 (0.15 %)	6 (0.29 %)	3 (0.36 %)	18 (4.1 %)
Permanent	4 (0.31 %)	7 (0.34 %)	3 (0.36 %)	1 (0.22 %)
Total	6	9	10	19
Hypoparathyroidism				
Temporary	18 (1.38 %)	30 (1.45 %)	33 (4.00 %)	71 (16.17 %)
Permanent	0 (0)	0 (0)	1 (0.12 %)	0 (0)
Total	18	30	34	71

*RLN* recurrent laryngeal nerve, *0* goitre that cannot be touched or seen, *I* goitre that can be touched but cannot be seen, *II* goitre that can be both touched and seen but is not beyond the exterior margin of the sternocleidomastoid muscle, *III* goitre that can be both touched and seen but is beyond the exterior margin of the sternocleidomastoid muscle

A total of 33 patients with solitary nodule or lesions with one lobe based on the US diagnosis were found to have micropapillary cancer in the contralateral lobe postoperatively.

## Discussion

The availability of levothyroxine replacement therapy makes patients who underwent TT, an iatrogenic thyroid absence, survive with euthyroid function, such that TT is a feasible management strategy for thyroid diseases. There is an increase in the utilisation of TT worldwide for the management of thyroid diseases [9, 15–18], although controversy remains for benign thyroid disorders and low-risk thyroid cancers. However, the significant role of this procedure is clear. Because thyroid diseases are rapidly increasing, we have attempted to establish an appropriate practice for thyroid disorders in economically underdeveloped areas, and the establishment of these practices is based on concepts, principles, practical experience and patient preference.

### Total thyroidectomy versus thyroid lobectomy for low-risk papillary thyroid carcinoma

Surgery is the basic treatment for thyroid malignancy, but for low-risk papillary thyroid cancer, there has been a long unsolved debate about the extent of primary surgery [6]. Papillary thyroid cancer covers the majority of thyroid cancers and has a favourable long-term survival rate [19–21]. The 10-year relative survival rate for patients with papillary thyroid cancer was 93 % for those treated in the USA [22]. The excellent disease-specific survival rates of low-risk thyroid cancer and the possible surgical complications associated with a more extensive approach make HT a preferred treatment for patients with a solitary nodule or lesions within one lobe according to some experts. The controversy focuses on the following points: (a) Does the bilateral procedure promote the overall survival rates? (b) Is the recurrence rate decreased by a more extensive surgery? (c) Compared to HT, is there an increase of complication rates associated with TT? (d) Can removing all thyroid tissue provide an adjuvant therapy and/or less complicated monitoring for recurrence disease? (e) Can the cost-effectiveness and quality of life be improved with a thorough surgical procedure?

In some retrospective studies, the mortality rates for low-risk well-differentiated thyroid cancer were extremely low, even during a relatively long follow-up period regardless of the extent of the initial surgery [23–25]. However, because of the absence of randomised prospective trials and a long enough follow-up time, it remains unclear whether there is a significant difference in the survival rates between HT and TT performed for patients with low-risk thyroid carcinoma. The disease recurrence rates are lower with TT in most studies [20, 23, 26, 27]. Although the recurrent diseases may be excised without increasing the complications and without affecting survival, an extra medical cost is incurred, including medical examination and reoperation, and the psychological burden of the fear of progression of malignant disease is not assessable as a pecuniary loss. Biological characteristics of papillary cancer are bilateral and multifocal [28, 29], and this matches our experience. In view of such an understanding, TT can remove all foci to the maximum extent. Complications associated with thyroid surgery are certainly a treatment consideration. Choosing HT for primary surgical treatment may be associated with lower complication rates [30, 31]; however, considering the trend towards lower morbidity when performed by experienced surgeons and the high risk of complication rates of completion thyroidectomy for recurrent diseases, the bilateral procedure as an initial treatment is a wise choice. Furthermore, TT will facilitate radioactive iodine ablation treatment for adjuvant therapy and serum thymoglobulin (Tg) level monitoring for disease recurrence. For the initial surgical treatment, TT dominates over HT as the most cost-effective surgical management for low-risk thyroid carcinoma, and this approach maximises the quality-adjusted life expectancy in patient PTC compared to lobectomy [32].

### Total thyroidectomy versus subtotal thyroidectomy for benign thyroid diseases

TT is controversial for low-risk malignant diseases and benign thyroid diseases. Compared with ST, in recent years, TT is increasingly utilised as the first-time surgical treatment for benign bilateral thyroid diseases, including Graves’ disease and toxic and non-toxic multinodular goitres [7, 9, 15–17]. However, understanding and applying this approach remains difficult in our locality, and bilateral subtotal thyroidectomy (BST) was the principal procedure for bilateral benign thyroid disease until now. Compared to ST, the extent of TT involves two extremely critical and vulnerable structures, the RLN and the parathyroid, and the related complications are the major deterrent to surgeons performing this approach. For ST, leaving a small portion of thyroid tissue in situations where it protects RLNs and the parathyroid from the injury of extensive manipulation seems to reduce morbidity rates compared to TT. In fact, the incidence of permanent morbidity after initial TT is reportedly low and is comparable to ST [9, 33]. Currently, it is unequivocally acknowledged that the routine exposure of RLN significantly reduces the risk of RLN injury [24, 34]. Furthermore, parathyroid autotransplantation is widely used and avoids the risk of permanent hypoparathyroidism [35, 36]. Additionally, remnant thyroid tissues, following ST for multinodular goitre, have a high percentage of micronodule formation with remarkable cellular proliferative activity [37, 38]. The recurrence rates of bilateral multinodular goitre after ST are significantly higher than for those who underwent TT, and the recurrence rates remain high even with treatment of levothyroxine suppression, which was once advocated to control the progression of benign thyroid nodules and prevent recurrence after partial thyroid surgery [9, 39, 40]. Thyroid surgery provides an effective and immediate solution for Graves’ disease, and it improves the quality of life for patients [41]. Compared to ST, TT is associated with a reduced incidence of recurrent hyperthyroidism and should be proposed for the treatment for Graves’ disease [8, 42]. In our surgical experience of first-time thyroidectomy, 879 patients had intraoperatively nonpalpable lesions (101 malignant and 778 benign) nearby the berry ligament, which remains when ST is performed instead of TT. As a consequence, removing all thyroid tissue for the first time essentially eliminates the risk of developing recurrent disease and thyroid carcinoma in the remnant tissue without an accompanying increased risk of permanent hypoparathyroidism or RLN injury.

The association of Hashimoto’s thyroiditis and thyroid cancer warrants a thorough examination for patients with thyroid nodules [43–45]. Therefore, we propose that TT should be performed for patients with thyroid nodules and this hypothesis should be tested by clinical controlled trials.

The avoidance of permanent hypoparathyroidism and bilateral RLN palsy is of prime importance, and the complications from these may be detrimental to the physical and mental health of the patient rather than the thyroid diseases themselves. During the identification and protection of the RLN and parathyroid intraoperatively, there is an increase in the temporary complication rates occurring in patients with giant thyroid goitre and Graves’ disease in our experience of first-time surgery. Our preliminary analysis suggests that gland goitre compression and inflammatory conglutination make it difficult to separate and reserve the RLN and parathyroid smoothly without any further stretching. Nevertheless, because there is no increase in the risk of permanent complications, we propose that such a transient injury of the RLN and parathyroid is controllable, and in our opinion, although this practice is difficult, the RLN and parathyroid can be preserved. However, maintaining the RLN and parathyroid during the revision surgery after ST is a different situation. Because of scar tissue and adhesion formation, the normal anatomical form, position and space of the adjacent tissue are obscured and injury of the RLN and parathyroid usually occurs without any warning intraoperatively. Therefore, no matter how meticulously the surgery was performed, even by experienced surgeons, the incidence of permanent RLN palsy and hypoparathyroidism of secondary surgery remains at a high level. The same result was reported in many studies [46–48]. Revision surgery for BST patients will not only increase the risk to patients but also increase the psychological pressure for the surgeons performing the procedures. In conclusion, a thorough initial surgical treatment should be adopted to avoid any potential and additional risks of reoperative surgery for recurrent diseases, and we suggest ‘*one side*, *once operation*’, which recommends that if lesion is within one lobe, then total lobectomy should be performed, and if the thyroid disorder is diffuse or involves two sides, total thyroidectomy should be performed. Repeat thyroid surgery for recurrent goitre after hemithyroidectomy for unilateral lesion does not carry an increased risk if the contralateral side has been left untouched.

### Patient preferences

Evidence-based medicine integrates the patient preferences with clinical experience and the best research evidence to make optimal medical decisions. Thus, when the patient is well informed of all implications and on the prerequisite of not violating the principle medical care, the patient’s wishes should be fully respected. Generally, patient preferences depend on the following factors for the treatment of thyroid diseases: (a) cancerisation; (b) recurrence; (c) cost, including examination, surgery, medical therapy and re-examination; (d) psychological burden; (e) lifelong levothyroxine replacement; (f) tolerance of medical therapies and associated side effects, such as anti-thyroid gland medicine; (g) education level; (h) income; and (i) medical insurance. Local patients who were subject to economic factors prefer a procedure that cures the diseases once and for all because such a solution may minimise the cost of postoperative management and examination and surgery for recurrent disease. Furthermore, compared to lifelong replacement therapy, repetitive examination because of the fear of recurrent disease will not only increase the cost but also aggravate the psychological burden. As an immediate and permanent cure, TT is a more worthwhile and necessary approach for most patients in our locality. Therefore, in an economically less-developed region, TT coincides better with patient preferences, at least in our experience.

A major drawback of this study is the lack of a thorough long-term follow-up; therefore, we cannot discuss the issue with data for the long-time survival rate of malignant thyroid diseases (particularly low-risk thyroid cancer) and recurrent disease. Another limitation is that all surgeries were performed or were under the supervision of the same experienced surgeon (Linfeng Wu), and this may cause a subjective preference and personal limitation for management for thyroid diseases, although our clinical decisions were based on evidence-based medicine. Furthermore, the small sample size of the secondary surgery group compared to the first-time surgery group must be considered.

## Conclusions

TT is an effective and safe approach based on increasing evidence. To prevent the hard-to-reduce prevalence of permanent complications of repeat surgery for recurrent disease, we prefer TT as an initial treatment for Graves’ disease and multinodular goitre when both lobes are involved. Because of the significantly lower rate of recurrence and no need for secondary surgery after malignant disease is confirmed by FNA or intraoperative frozen section, TT is performed whether the contralateral lobe is involved. Furthermore, the core principle of all surgical management of thyroid diseases is ‘*one side once operation*,’ and this principle should be widely disseminated to deepen the understanding that the true essence of thyroidectomy is the safety of the RLN and parathyroid gland. Therefore, in response to the rapid increase of thyroid diseases in less-developed regions, TT may be considered the most reasonable treatment of choice for patients with thyroid disorders.
